# The image-based preoperative fistula risk score (preFRS) predicts postoperative pancreatic fistula in patients undergoing pancreatic head resection

**DOI:** 10.1038/s41598-022-07970-2

**Published:** 2022-03-08

**Authors:** Fiona R. Kolbinger, Julia Lambrecht, Stefan Leger, Till Ittermann, Stefanie Speidel, Jürgen Weitz, Ralf-Thorsten Hoffmann, Marius Distler, Jens-Peter Kühn

**Affiliations:** 1 Department of Visceral, Thoracic and Vascular Surgery, University Hospital and Faculty of Medicine Carl Gustav Carus, Technische Universität Dresden, TU Dresden, Fetscherstrasse 74, 01307 Dresden, Germany; 2 Division of Translational Surgical Oncology, National Center for Tumor Diseases (NCT), Partner Site Dresden, Dresden, Germany; 3 Else Kröner Fresenius Center for Digital Health (EKFZ), Technische Universität Dresden, Dresden, Germany; 4 Institute and Policlinic for Diagnostic and Interventional Radiology, University Hospital Carl Gustav Carus Dresden, TU Dresden, Fetscherstrasse 74, 01307 Dresden, Germany; 5 Institute for Community Medicine, University Medicine Greifswald, Greifswald, Germany

**Keywords:** Outcomes research, Surgical oncology, Computed tomography, Pancreatic cancer, Pancreatitis

## Abstract

Clinically relevant postoperative pancreatic fistula (CR-POPF) is a common severe surgical complication after pancreatic surgery. Current risk stratification systems mostly rely on intraoperatively assessed factors like manually determined gland texture or blood loss. We developed a preoperatively available image-based risk score predicting CR-POPF as a complication of pancreatic head resection. Frequency of CR-POPF and occurrence of salvage completion pancreatectomy during the hospital stay were associated with an intraoperative surgical (sFRS) and image-based preoperative CT-based (rFRS) fistula risk score, both considering pancreatic gland texture, pancreatic duct diameter and pathology, in 195 patients undergoing pancreatic head resection. Based on its association with fistula-related outcome, radiologically estimated pancreatic remnant volume was included in a preoperative (preFRS) score for POPF risk stratification. Intraoperatively assessed pancreatic duct diameter (*p* < 0.001), gland texture (*p* < 0.001) and high-risk pathology (*p* < 0.001) as well as radiographically determined pancreatic duct diameter (*p* < 0.001), gland texture (*p* < 0.001), high-risk pathology (*p* = 0.001), and estimated pancreatic remnant volume (*p* < 0.001) correlated with the risk of CR-POPF development. PreFRS predicted the risk of CR-POPF development (AUC = 0.83) and correlated with the risk of rescue completion pancreatectomy. In summary, preFRS facilitates preoperative POPF risk stratification in patients undergoing pancreatic head resection, enabling individualized therapeutic approaches and optimized perioperative management.

## Introduction

Postoperative pancreatic fistula (POPF) is one of the most common severe complications after pancreatic surgery, typically resulting from leakage or insufficiency of the pancreato-enteric anastomosis^1^. POPF affects up to 30% of patients undergoing pancreaticoduodenectomy, with highly varying numbers depending on a surgical center’s size, expertise and follow-up duration^2–4^.

According to the 2016 update of the International Study Group on Pancreatic Surgery (ISGPS), POPF is, depending on laboratory parameters and clinical presentation, classified into biochemical leak (formerly termed grade A POPF), grade B and C POPF, the latter two causing prolongation of inpatient treatment (clinically relevant POPF, CR-POPF). Grade C POPFs are a potentially life-threatening complication characterized by single or multiple organ failure, typically necessitating intensive care as well as surgical revision^2^. The reported mortality of a grade C POPF is 44%^5^. Because of otherwise unmanageable complications of pancreatic surgery, mostly POPF and post-pancreatectomy hemorrhage, rescue completion (total) pancreatectomy can be necessary as a last-resort therapeutic option in cases, in which pancreas-preserving treatment options are technically unfeasible. This procedure is characterized by exceptionally high morbidity and in-hospital mortality of over 40%^6,7^.

While some existing pancreatic fistula risk scores (FRSs) reliably predict an increased risk of POPF development in large patient cohorts^3,8^, most of these scores rely on intraoperatively assessed factors such as palpated texture of pancreatic parenchyma or estimated blood loss, thus allowing for risk stratification only during the operation. In a clinical setting however, preoperative risk stratification is required to facilitate consideration of the risk of POPF development already during the planning phase of pancreatic surgery. This would allow for critical reassessment of alternative surgical and conservative treatment approaches in patients with a preoperative high-risk constellation. Cross-sectional imaging such as contrast-enhanced computed tomography (CT) is the clinical standard modality for diagnosis of pancreatic pathologies and is commonly used for pancreatic surgery planning. Moreover, CT provides information about pancreatic texture, pancreatic size, and duct diameter. Therefore, preoperative CT could serve as the basis of a preoperative image-guided FRS.

This study aims to explore and assess the value of preoperative CT-derived risk factors in predicting an increased risk of POPF development, aiming to facilitate preoperative patient stratification as well as a POPF risk-adapted surgical approach and optimized perioperative management through a preoperatively available risk score.

## Results

### Patient characteristics

Between 09/2012 and 11/2021, 195 patients (82 female and 113 male, mean age 67.3 ± 10.3 years) undergoing pancreatic head resection were included in this study (Table 1). All patients had a clinical indication for the operation (163 tumor, 17 chronic pancreatitis, 10 cystic neoplasia, 5 other). Most patients (n = 147) underwent pylorus-preserving pancreaticoduodenectomy, Whipple procedure was performed in 48 cases.

**Table 1:** Patient characteristics. Mean age is displayed in years (± SD), for sex, indication and surgery type, total numbers and proportion of the cohort are displayed.

	Total	POPF occurrence			Rescue completion pancreatectomy
		No CR-POPF	Grade B	Grade C	
Age (years)	67.3 ± 10.3	66.8 ± 10.4	67.7 ± 8.4	69.4 ± 11.8	69.6 ± 9.7
**Sex**					
Female	82 (42.0%)	60 (30.8%)	13 (6.7%)	9 (4.6%)	4 (2.1%)
Male	113 (58.0%)	79 (40.5%)	17 (8.7%)	17 (8.7%)	10 (5.1%)
**Indication**					
Tumor	163 (83.6%)	111 (56.9%)	28 (14.4%)	24 (12.3%)	13 (6.7%)
Chronic pancreatitis	17 (8.7%)	17 (8.7%)	0	0	0
Cystic neoplasia	10 (5.1%)	7 (3.6%)	1 (0.5%)	2 (1.0%)	1 (0.5%)
Other	5 (2.6%)	4 (2.1%)	1 (0.5%)	0	0
**Surgery type**					
Pylorus-preserving pancreaticoduodenectomy	147 (75.4%)	109 (55.9%)	21 (10.8%)	17 (10.8%)	8 (4.1%)
Whipple	48 (24.6%)	30 (15.4%)	9 (4.6%)	9 (4.6%)	6 (3.1%)

*POPF* postoperative pancreatic fistula.

During the clinical course, CR-POPF occurred in 56 patients. Out of these patients, 30 and 26 patients suffered from grade B and grade C POPF, respectively. A total of 139 patients did not develop CR-POPF. Surgical complications following pancreatic head resection necessitated total pancreatectomy in 14 patients. The most prevalent indications for this procedure were hemorrhage (n = 7), fulminant (remnant) pancreatitis (n = 4), or a combination of both (n = 3).

### Image-based rFRS correlates with intraoperatively assessed sFRS and fistula-related outcome in patients undergoing pancreatic head resection

**Figure 1:**
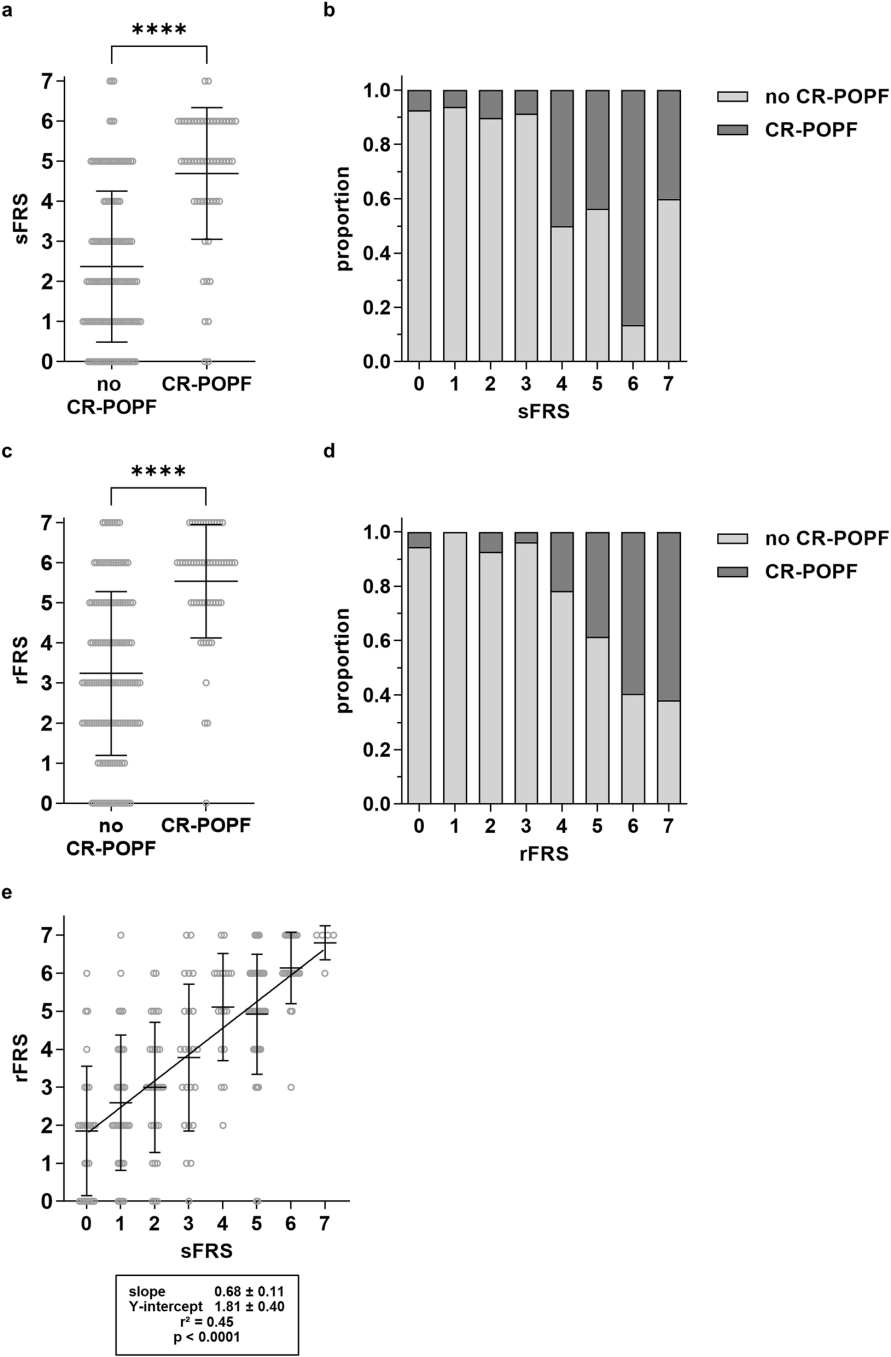
Image-based rFRS correlates with intraoperatively assessed sFRS and fistula-related outcome in patients undergoing pancreatic head resection. (**a**) Mean sFRS in relation to manifestation of CR-POPF. **(b)** Manifestation of CR-POPF in relation to sFRS. **(c)** Mean rFRS in relation to manifestation of CR-POPF. **(d)** Manifestation of CR-POPF in relation to rFRS. **(e)** Correlation of preoperative rFRS with intraoperative sFRS. Horizontal lines and error bars represent mean values and SD, respectively. Symbols represent individual patients and bars represent patient distribution. Statistical analysis was performed using unpaired, two-tailed *t*-test (****: *p* < 0.0001; ***: 0.0001 < *p* < 0.001; **: 0.001 ≤ *p* < 0.01; *: 0.01 ≤ *p* < 0.05, ns: not significant). Abbreviations: clinically relevant postoperative pancreatic fistula (CR-POPF).

All three individual sFRS parameters, intraoperatively assessed pancreatic texture (*p* < 0.001), pathology (*p* < 0.001), and pancreatic duct diameter (3.05 mm ± 1.58 mm and 4.14 mm ± 1.88 mm in patients with and without CR-POPF, respectively, *p* < 0.001), independently predicted the risk of CR-POPF development (Table 2). The sFRS was significantly higher in patients who developed CR-POPF during the clinical course (4.70 ± 1.64) than in patients who did not (2.37 ± 1.88, *p* < 0.0001, Fig. 1a). Moreover, intraoperatively determined sFRS correlated with manifestation of CR-POPF in our patient cohort, with CR-POPF occurring in more than 50% of patients with an sFRS of 4 or more (Fig. 1b).

**Table 2:** Association between sFRS and rFRS risk factors as well as additional image-based parameters and CR-POPF occurrence.

Risk factor	No CR-POPF	CR-POPF	*p*-value
**sFRS**			
Pancreatic texture			< 0.001
Hard	89 (45.6%)	6 (3.1%)	
Soft	50 (25.6%)	50 (25.6%)	
Pathology			< 0.001
Low-Risk	89 (45.6%)	13 (6.7%)	
High-Risk	50 (25.6%)	43 (22.0%)	
Pancreatic duct diameter (mm)	4.14 ± 1.88	3.05 ± 1.58	< 0.001
≥ 5 mm	42 (21.5%)	7 (3.6%)	
≥ 4 mm	40 (20.5%)	6 (3.1%)	
≥ 3 mm	35 (18.0%)	17 (8.7%)	
≥ 2 mm	18 (9.2%)	24 (12.3%)	
< 2 mm	4 (2.1%)	2 (1.0%)	
**rFRS**			
Pancreatic texture			0.001
Atrophic	37 (19.0%)	1 (0.5%)	
Normal	102 (52.3%)	55 (28.2%)	
Pathology			< 0.001
Low-Risk	92 (47.2%)	22 (11.3%)	
High-Risk	47 (24.1%)	34 (17.4%)	
Pancreatic duct diameter (mm)	4.78 ± 2.59	2.56 ± 1.46	< 0.001
≥ 5 mm	55 (28.2%)	4 (2.1%)	
≥ 4 mm	22 (11.3%)	3 (1.5%)	
≥ 3 mm	25 (12.8%)	2 (1.0%)	
≥ 2 mm	21 (10.8%)	29 (14.9%)	
< 2 mm	16 (8.2%)	18 (9.3%)	
Normalized pancreatic density	1.49 ± 0.63	1.56 ± 0.77	0.56
Estimated PRV (cm^3^)	24.7 ± 14.5	37.6 ± 13.6	< 0.001

Low-risk pathology encompasses pancreatic ductal adenocarcinoma and chronic pancreatitis, and high-risk pathology comprises all other histopathological entities.

*CR-POPF* clinically relevant postoperative pancreatic fistula, *PRV* pancreatic remnant volume.

Correlation of rFRS with the manifestation of CR-POPF revealed a similar pattern as had been observed with sFRS: all three individual rFRS risk factors, radiographically determined pancreatic texture (*p* < 0.001), pathology (*p* = 0.001), and pancreatic duct diameter (2.56 mm ± 1.46 mm and 4.78 mm ± 2.59 mm in patients with and without CR-POPF, respectively, *p* < 0.001), significantly correlated with the risk of CR-POPF development (Table 2). Of note, pancreatic texture and pancreatic duct diameter were independently and blindly rated by an expert radiologist, resulting in substantial inter-rater agreement (Cohen’s kappa of 0.75 and 0.63 for pancreatic texture and pancreatic duct diameter, respectively). Total rFRS was significantly lower in patients who did not develop CR-POPF (3.24 ± 2.04) than in patients who developed CR-POPF (5.54 ± 1.41) during the postoperative course (*p* < 0.001, Fig. 1c). Moreover, high preoperative rFRS was predictive of high risk of CR-POPF development in the analyzed patient cohort: at an rFRS up to 4, the risk of CR-POPF development was below 20%, whereas more than 50% of patients with an rFRS of 5 or more developed CR-POPF (Fig. 1d). CR-POPF prediction accuracies of sFRS and rFRS were similar at AUCs of 0.82 and 0.82, respectively. Comparison of sFRS and rFRS revealed a linear correlation between both scores (r^2^ = 0.45, *p* < 0.0001, Fig. 1e). Normalized pancreatic density did not show significant statistical correlation with the risk of CR-POPF development, while estimated PRV did (Table 2 and Supplementary Table 1). Besides these parameters, body-mass index (*p* = 0.001), and the absence of diabetes prior to surgery (*p* = 0.005) significantly correlated with the occurrence of CR-POPF, while age (*p* = 0.47), sex (*p* = 0.619), and surgical approach (pylorus-preserving pancreaticoduodenectomy or Whipple surgery, *p* = 0.121) did not.

According to TRIPOD recommendations^9^, we additionally performed a leave-one-out cross-validation to assess the prediction performance of both prediction models’ risk factors (sFRS and rFRS parameters), confirming the abovementioned results (Supplementary Table 1).

### Preoperatively estimated pancreatic remnant volume correlates with the risk of CR-POPF development

**Figure 2:**
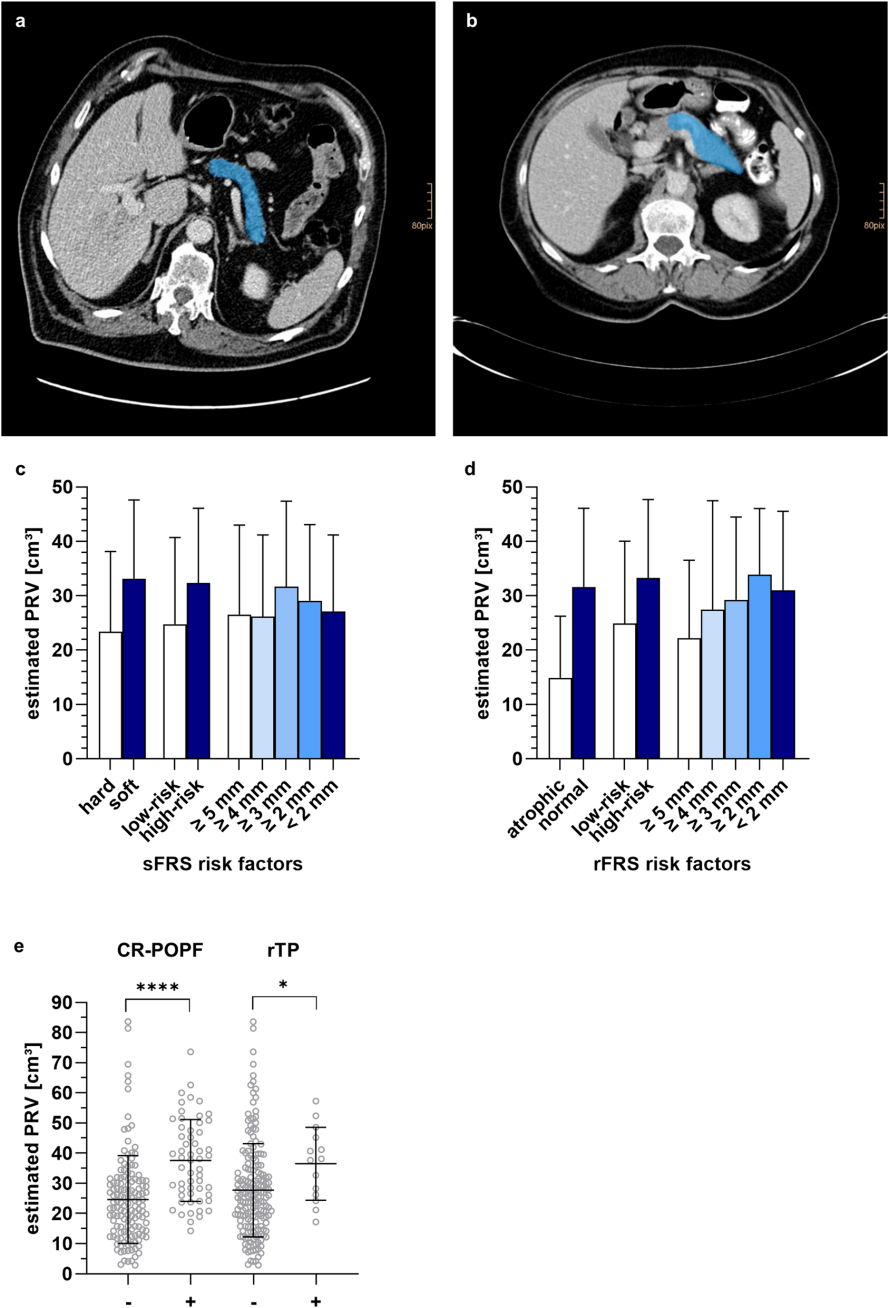
Preoperatively estimated pancreatic remnant volume correlates with the risk of CR-POPF development. (**a**,**b**) Example illustration of PRV estimation in axial reconstructions of preoperative contrast-enhanced CT images. Blue segmentations represent the anticipated remaining pancreas. **(c,d)** Mean estimated PRV in relation to manifestation of sFRS **(d)** and rFRS **(d)** risk factors. **(e)** Mean estimated PRV in relation to occurrence of CR-POPF and rescue total pancreatectomy during the clinical course. Symbols, bars or horizontal lines and error bars represent individual patients, mean values and SD, respectively. Statistical analysis was performed using unpaired, two-tailed *t*-test (****: *p* < 0.0001; ***: 0.0001 < *p* < 0.001; **: 0.001 ≤ *p* < 0.01; *: 0.01 ≤ *p* < 0.05, ns: not significant). Abbreviations: clinically relevant postoperative pancreatic fistula (CR-POPF), pancreatic remnant volume (PRV), rescue completion (total) pancreatectomy (rTP).

On average, preoperatively estimated PRV (Fig. 2a,b) showed a marked association with high-risk sFRS and rFRS features such as soft pancreatic texture, high-risk pathology and small pancreatic duct diameter in our patient cohort (Fig. 2c,d). Moreover, estimated PRV predicted the risk of CR-POPF development (*p* < 0.001), and patients undergoing rescue completion (total) pancreatectomy during the clinical course following pancreatic head resection had significantly higher preoperatively estimated PRV than patients, in which this high-risk procedure was not necessary (*p* = 0.04, Fig. 2e). In the 153 cases, in which postoperative CT images were available, preoperatively estimated PRV displayed good correlation with actual PRV (Supplementary Fig. 3).

In summary, our results demonstrate that preoperative CT-based volumetry can approximate actual PRV. Estimated PRV correlates with the manifestation of CR-POPF as well as high-risk pathology, soft pancreatic texture and small pancreatic duct diameter.

### High preFRS preoperatively predicts an increased risk of CR-POPF as well as rescue completion pancreatectomy during the clinical course

**Figure 3:**
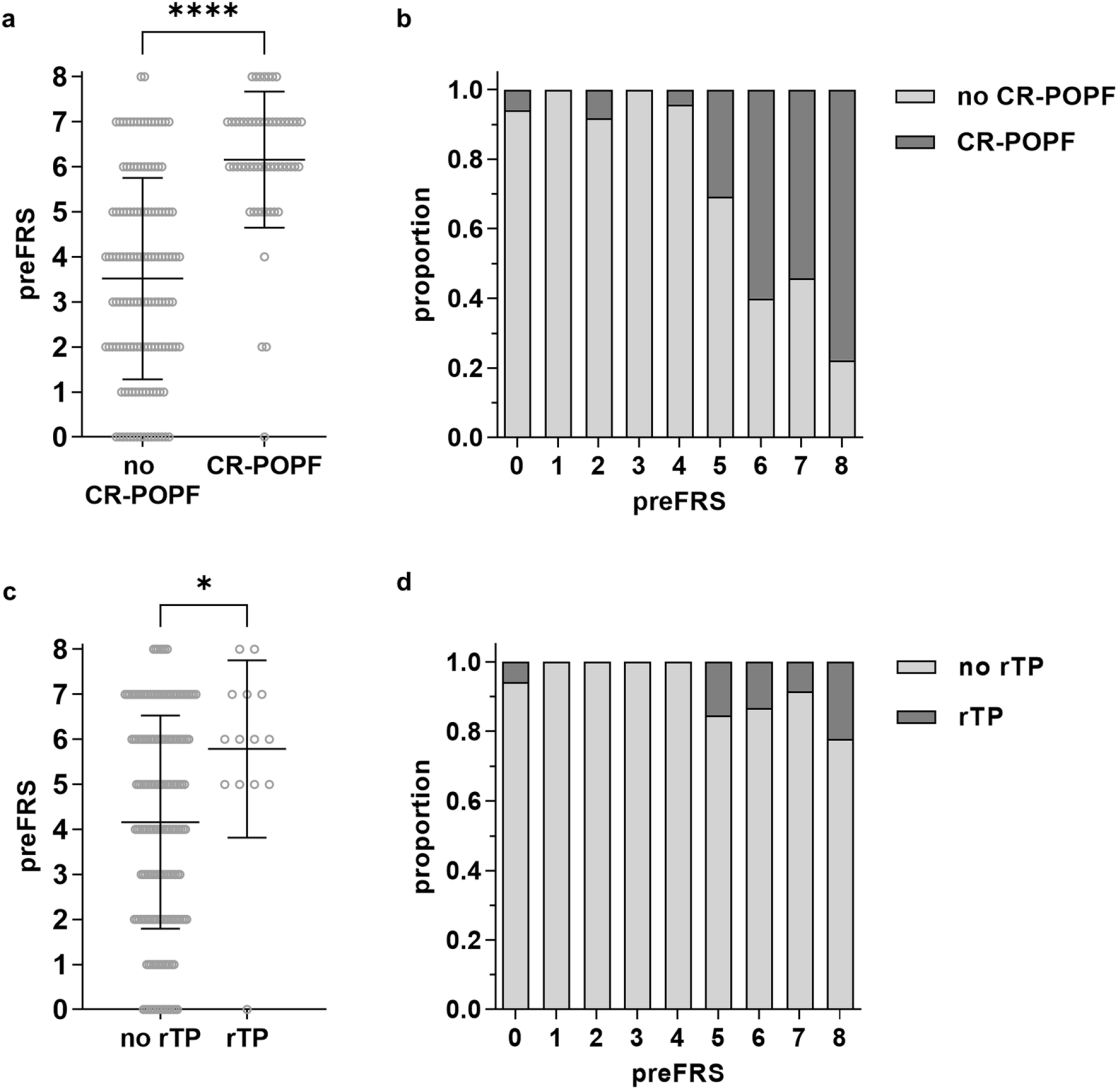
Preoperative preFRS correlates with fistula-related outcome and an increased risk of rescue completion (total) pancreatectomy in patients undergoing pancreatic head resection. (**a**) Mean preFRS in relation to manifestation of CR-POPF. **(b)** Manifestation of CR-POPF in relation to preFRS. **(c)** Mean preFRS in relation to the indication of rescue completion (total) pancreatectomy during the clinical course. **(d)** Indication of rescue completion (total) pancreatectomy during the clinical course in relation to preFRS. Horizontal lines and error bars represent mean values and SD, respectively. Symbols represent individual patients and bars represent patient distribution. Statistical analysis was performed using unpaired, two-tailed *t*-test (****: *p* < 0.0001; ***: 0.0001 < *p* < 0.001; **: 0.001 ≤ *p* < 0.01; *: 0.01 ≤ *p* < 0.05, ns: not significant). Abbreviations: clinically relevant postoperative pancreatic fistula (CR-POPF), rescue completion (total) pancreatectomy (rTP).

Based on the identified value of preoperatively estimated PRV in predicting CR-POPF, this risk factor was included in a preoperative FRS (preFRS) based entirely on standard preoperative imaging (Table 3b). PreFRS was significantly higher in patients who developed CR-POPF during the postoperative course (6.16 ± 1.51) than in patients who did not (3.52 ± 2.23, Fig. 3a). CR-POPF manifested in over 60% of patients with a preFRS of 6 or more (Fig. 3b), and in leave-one-out cross validation, preFRS displayed good accuracy in prediction of CR-POPF at an AUC of 0.83 (Supplementary Table 1). Additional consideration of these preoperatively available clinical features resulted in no further improvement of the model on the validation folds (Supplementary Table 1).

**Table 3:** Surgical and image-based parameters contributing to sFRS and rFRS (a) as well as preFRS (b).

Risk factor	Parameter (sFRS) (surgeon’s intraoperative assessment)	Parameter (rFRS) (assessment of preoperative contrast-enhanced CT)	Points
**a**			
Pancreatic texture	Hard	Atrophic	0
	Soft	Normal	2
Pathology	Ductal adenocarcinoma, chronic pancreatitis		0
	Other (ampullary, duodenal, cholangiocellular, or islet cell carcinoma, metastasis, etc.)		1
Pancreatic duct diameter	≥ 5 mm		0
	≥ 4 mm		1
	≥ 3 mm		2
	≥ 2 mm		3
	< 2 mm		4
			Total: 0–7 points

Risk factor	Parameter (preFRS) (assessment of preoperative contrast-enhanced CT)	Points	
**b**			
Pancreatic texture	Atrophic	0	
	Normal	2	
Pathology	Ductal adenocarcinoma, chronic pancreatitis	0	
	Other (ampullary, duodenal, cholangiocellular, or islet cell carcinoma, metastasis, etc.)	1	
Pancreatic duct diameter	≥ 5 mm	0	
	≥ 4 mm	1	
	≥ 3 mm	2	
	≥ 2 mm	3	
	< 2 mm	4	
Estimated PRV	≤ 30 mL	0	
	> 30 mL	1	
		Total: 0–8 points

Out of the analyzed 195 patients, 56 patients developed CR-POPF, and 14 patients underwent rescue completion pancreatectomy during the clinical course following pancreatic head resection (Table 1). In our patient cohort, patients undergoing rescue completion pancreatectomy had significantly higher preFRS (5.79 ± 1.97) than patients not requiring this high-risk procedure during the postoperative course (4.16 ± 2.37, Fig. 3c). Out of 14 patients undergoing rescue completion pancreatectomy following pancreatic head resection, 13 had a preFRS of at least 5, and grade C POPF was present in 13 of these patients (Fig. 3d).

## Discussion

In summary, the presented data demonstrate that risk factors for the development of CR-POPF, such as small pancreatic duct diameter and soft pancreatic texture, can be determined before surgery based on preoperative contrast-enhanced CT imaging. Four CT-derived parameters individually markedly correlated with the risk of CR-POPF development in our cohort: normal (non-atrophic) pancreatic morphology, small pancreatic duct diameter, radiologically assessed high-risk pathology and high estimated PRV. These factors were integrated into the preFRS, a highly clinically applicable image-based risk score for preoperative patient risk stratification, offering a prediction accuracy comparable to the consideration of clinically accepted intraoperatively assessed POPF risk factors.

One of the most commonly used intraoperative POPF risk stratification systems, the pancreatic fistula risk score (FRS) proposed by Callery et al*.* in 2013^3^, incorporates the risk factors for POPF development recognized by the International Study Group of Pancreatic Fistula: small pancreatic duct diameter, soft pancreatic texture, high-risk pathology and excessive intraoperative blood loss. The value of these variables, however, is controversially discussed, and association analyses have demonstrated differential relevance and significance of the contributing parameters^10,11^. While soft pancreatic texture and small pancreatic duct diameter are highly predictive of POPF development, recent studies imply no relevant association of high-volume intraoperative blood loss with a significantly increased risk of POPF development^8,10–13^. Based on this evidence and aiming at an integration of preoperatively available parameters, blood loss was not considered as a potential predictor in this analysis.

To date, few studies have analyzed the value of preoperative CT images in predicting POPF, and most of these studies have focused on individual image-based parameters. In line with the presented results, image-based pancreatic duct diameter and estimated PRV have been found to be associated with the risk of POPF development in patients undergoing pancreaticoduodenectomy^14–16^. In addition to pancreatic duct diameter and estimated PRV, pancreatic morphology and image-based pathology strongly correlated with the risk of CR-POPF development in our patient cohort. The image-based preFRS is based on the four abovementioned parameters and showed comparable accuracy in predicting CR-POPF as the intraoperatively assessed sFRS. In comparison to Mungroop et al*.*^8,17^, body-mass index, but not male sex correlated with the risk of POPF development in our cohort.

Practically, these results imply that standard-of-care preoperative imaging can facilitate an integration of anticipated POPF risk into the planning phase of pancreatic surgery. Particularly in patients with pancreatic cancer, the most prevalent indication for pancreatic head resection, preoperative identification of high-risk pancreato-enteric anastomosis could offer a significant advantage, as CR-POPF may cause a significant delay or suspension of adjuvant therapy, ultimately reducing chances for long-term survival^18–20^. Therefore, the preFRS could be used to identify high-risk patients that could profit from a more extensive procedure that completely avoids pancreato-enteric anastomosis and thus the risk of POPF development. In that respect, recent publications have explored the potential benefits of total pancreatectomy as opposed to pancreatic head resection^4,21,22^. While total pancreatectomy remains associated with substantial morbidity and mortality, works by Loos et al*.*^23^ and Hempel et al*.*^24^ provide strong evidence that total pancreatectomy is a safe surgical option, which can offer benefits in selected patients with high-risk pancreatic anastomosis. Other possible management options for patients with a high risk of pancreatic fistula include a variety of anastomotic techniques such as prophylactic splinting of the main pancreatic duct or reconstruction via pancreaticogastrostomy^25^.

Importantly, no clear recommendations exist with regard to an individualization of surgical drain placement and postoperative management aiming at a reduction of the CR-POPF rate. A recent meta-analysis identified an association of drain placement with lower mortality, but higher rates of CR-POPF after pancreaticoduodenectomy as compared to patients who did not receive an intraperitoneal drain^26^. Postoperative administration of somatostatin analogues has similarly demonstrated no clear benefit (but also no clear disadvantage) and remains controversial^27^. In the light of this controversy, both drain placement and postoperative administration of somatostatin analogues (Somatostatin, Pasireotide, Octreotide) were indicated on discretion of the operating surgeon in our cohort.

Technically, image-based preoperative assessment of the pancreatic duct diameter offers higher accuracy than intraoperative measurement, which is typically carried out through insertion of probes of integer millimeter thickness. This potentially causes an overestimation of the duct diameter, while radiological assessment is accurate to the second decimal of a millimeter. Especially in patients with very narrow duct diameters between 1 and 2 mm, the image-based assessment may therefore facilitate more exact measurement. This may be an explanation for the higher total number of patients in the presented cohort that have a pancreatic duct diameter of < 2 mm (n = 34) according to the radiological assessment as compared to the surgical assessment (n = 6).

The association of estimated PRV with the risk of CR-POPF development implies an interpretative hen-and-egg problem: on the one hand, low PRV might predominantly be a consequence of other low-risk features such as pancreatic atrophy and a dilated pancreatic duct, thus correlating with low CR-POPF risk. It is, however, also conceivable that a small pancreatic remnant may exert less exocrine function, resulting in a lower volume of pancreatic juice passing the pancreato-enteric anastomosis, consequently putting the anastomosis at less risk of insufficiency. In that respect, patients with exocrine insufficiency have been found to have an atrophic pancreas and a linear correlation between pancreatic volume and exocrine as well as endocrine activity of the pancreas has been identified^28^. This would support the theory that pancreatic volume is generally related to pancreatic morphology and function, yet the interplay and potential causalities between these features and the risk of CR-POPF development remain to be fully elucidated.

The limitations of this study are mostly related to its retrospective and monocentric character. We feel that despite the monocentric nature of the study, our analysis provides both interesting and valuable evidence about the value of preoperative imaging in facilitating anticipation of surgical complications in a robust cohort of 195 patients, out of which 56 patients developed a CR-POPF. The CR-POPF rate reported in this study is slightly above previously reported CR-POPF rates^2–4^, likely due to recruitment bias related to the retrospective character of the study. Moreover, institutional standards support a relatively early interventional treatment of POPF, potentially resulting in a tendency towards an overestimation of the proportion of patients with grade B POPF. As a further limitation, the CT images were not acquired in a standardized manner due to the retrospective nature of this study. Nevertheless, the variability of the images underlines the robustness of the presented results. Due to the lack of data on the perioperative development of endocrine and exocrine function, this study also cannot functionally elucidate the interplay between POPF risk factor manifestation, pancreatic (remnant) volume and exocrine as well as endocrine pancreatic function.

In conclusion, this study has identified an association between image-based pancreatic morphology, pancreatic duct diameter, pathology and estimated PRV with the risk of CR-POPF development. These factors were combined into the preFRS, a highly clinically applicable risk score predicting POPF risk at comparable accuracy as intraoperative evaluation of established risk factors. Based on the findings of this study, future research is needed for a prospective validation of the preFRS in an independent patient cohort. Ultimately, these investigations could result in the consideration of different therapeutic strategies in patients with a preoperative high-risk constellation (preFRS equal to or above 6). Specifically, primary total pancreatectomy may be taken into consideration as a therapeutic option in very selected cases with significantly impaired preoperative glucose tolerance or if concomitant islet cell autotransplantation is available at the surgical center.

## Methods

### Patient population and outcome variables

Between 09/2012 and 11/2021, a total of 195 patients undergoing pancreatic head resection (pylorus-preserving pancreaticoduodenectomy or Whipple procedure) at the University Hospital Carl Gustav Carus Dresden with available preoperative contrast-enhanced CT and documented intraoperatively assessed pancreatic duct diameter, pancreatic texture and histopathology were included in this retrospective study. All included patients had a clinical indication for the surgical procedure.

Frequency and severity of POPF were determined within a timeframe of 30 days after surgery or until discharge from hospital, whichever occurred last. Following International Study Group of Pancreatic Fistula standards, POPFs were classified according to their clinical relevance into BL, grade B and C POPF and subsequently summarized into “no CR-POPF” (no clinical signs of pancreatoenteric anastomotic leakage) and “CR-POPF” (grade B and C POPF)^2^. In addition, the frequency of salvage completion pancreatectomy during the clinical course of the pancreatic head resection was evaluated as an outcome variable.

This study was performed in accordance with all relevant guidelines and regulations, particularly with the Declaration of Helsinki and its later amendments. The local Institutional Review Board (Ethics Committee at the Technical University Dresden) reviewed and approved this study (approval number: BO-EK-263062020). The Ethics Committee at the Technical University Dresden waived the informed consent.

### Surgical FRS (sFRS)

Based on previous studies investigating risk factors for the development of CR-POPF^3,29^, intraoperatively documented pancreatic gland texture, pancreatic duct diameter and pathology were taken into account for determination of sFRS (Table 3). Intraoperative assessment of the surgical parameters (pancreatic texture and duct diameter) was carried out by at least two surgeons (primary surgeon and first assist) and the final documentation was made after discussion within the assessing surgical team.

According to the original FRS defined by McMillan et al., pancreatic texture was stratified into hard and soft. Intraoperative blood loss was not integrated into this score to facilitate association with preoperatively determinable parameters. The sum of the numerical values assigned to each of the 3 risk factors resulted in a sFRS between 0 (lowest risk of POPF development) and 7 (highest risk of POPF development) points (Table 3).

### Radiological FRS (rFRS) and preoperative FRS (preFRS)

Correspondingly, the rFRS was determined through assessment of pancreatic gland texture, pancreatic duct diameter and pathology (defined as the most likely suspected diagnosis) in preoperative contrast-enhanced CT images acquired for tumor staging purposes (Supplementary Fig. 1, Table 3a). Imaging was performed using the clinically available CT image datasets, which were obtained both in-house and externally. Due to the retrospective character of this study, no standardization with regard to hardware was performed. In general, only CT examinations with a slice thickness of less than 5 mm were included. All scans were acquired using an iodine-containing contrast agent with a delay corresponding to the portal venous phase.

CT images were blindly reviewed for pancreatic gland texture and pancreatic duct diameter by two radiologists with more than four years and more than 15 years of experience in abdominal CT imaging, respectively, using the picture archiving system PACS (Agfa Impax EE R20, Agfa Healthcare). Except for patient age and sex, all clinical parameters (in particular sFRS and clinical course) were blinded during assessment of the image-based parameters contributing to rFRS. Discrepancies for individual patients’ parameters were resolved through discussion. Pathology, pancreatic tissue density and pancreatic remnant volume were blindly assessed by a radiologist with more than four years of experience in abdominal CT imaging and reviewed by a radiological expert with more than 15 years of experience in abdominal CT imaging.

Pancreatic gland texture was classified as “atrophic” or “normal” based on CT images, and assigned partial scores of 0 and 2, respectively (Supplementary Fig. 2). The subjective evaluation of the texture parameters "atrophy" versus "normal" was performed by 2 experienced radiologists. Atrophy was defined as a loss of pancreas parenchyma with a parenchymal width measured from the main pancreatic duct wall of approximately 4 mm or less^30^. Pancreatic duct diameter (0–4 points) was measured via conventional distance measurement in the expected resection area in radiographically healthy pancreatic tissue immediately distal to the pancreatic head tumor (Table 3). Image-based assessment of the most likely pathology resulted in a partial score of 0 (adenocarcinoma or chronic pancreatitis) or 1 (other malignant entities including ampullary, duodenal, cholangiocellular or islet cell carcinoma, and pancreatic metastases of other malignancies). The sum of the numerical values allocated to each of the 3 risk factors was equivalent to the final rFRS (0–7).

Additionally, segmentation of healthy pancreatic tissue distal to the tumor served to assess both the expected volume of the postoperatively remaining pancreas and pancreatic tissue density (defined as the mean density of healthy pancreatic tissue distal to the tumor normalized to muscle density). The margins of the expected pancreatic remnant were set according to the surgical concept of circumferential resection margins with a desired distance of > 1 mm from suspected malignant foci of the pancreas. For inflammatory processes, margins were determined in the same manner. For additional consideration of the estimated pancreatic remnant volume (PRV), estimated PRVs equal or lower than 30 mL (75th percentile of patients not developing CR-POPF during the clinical course) and higher than 30 mL were assigned partial scores of 0 and 1, respectively (Table 3B). These partial scores were added to the rFRS, resulting in the final preFRS (0–8).

### Statistical analysis

Inter-rater reliability between the two independent reviewers was assessed using Cohen’s kappa^31^. Association of individual parameters of the sFRS and the rFRS (pancreatic texture, pathology and pancreatic duct diameter) as well as additional image-based parameters (estimated pancreatic remnant volume (PRV) and pancreatic tissue density) and the resulting sFRS and rFRS with the occurrence of CR-POPF were assessed by Wilcoxon (continuous data) and Chi^2^ tests (categorical data). The prediction accuracy of sFRS, rFRS and preFRS regarding occurrence of CR-POPF was evaluated using a logistic regression model. Simple linear regression was used to model for the relationship between sFRS and rFRS. Unpaired, two-tailed t-test served to compare FRS and estimated PRV in patients with and without CR-POPF as well as with and without rescue completion (total) pancreatectomy. We report continuous data as mean ± SD and categorical data as absolute number and percentage.

For the conducted experiments the in-house developed “Fully Automated Machine Learning with Interpretable Analysis of Results” (FAMILIAR) framework was used to train and validate the models based on sFRS features, rFRS features and a combination of clinical features (sex, body mass-index, presence of diabetes prior to surgery, age, surgery type) and rFRS features. The risk models were developed and validated as previously described^32–34^ using a leave-one-out cross-validation (LOOCV) scheme. Briefly, the computed features were transformed and normalized using Yeo-Johnson and the z-score methods, respectively. Afterwards, feature selection was performed based on the panelized logistic regression approach using 20 bootstrap samples of the training folds. Subsequently, we trained a logistic regression model on 20 bootstrap samples of the training folds, using the highest ranked features and an optimized signature size according to Hutter et al*.*^35^*.* Finally, an ensemble prediction was made by averaging the prediction scores of each model for both the training and validation fold separately. The model performance was assessed using the average area under the curve of the receiver-operator characteristic curve (AUC) and the F1 score. Furthermore, all evaluated features were tested on the entire cohort for a statistically significant association with the occurrence of CR-POPF using a two-sided two-sample t-test with Benjamini–Hochberg adjustment^36^ for multiple testing.

For statistical analysis and risk modelling, SPSS (SPSS Statistics version 26.0, IBM), GraphPad Prism (Version 9.0.0, GraphPad Software, LLC) and the R software package (R version 3.1.3, The R Foundation) were used. Continuous data are presented as mean ± standard deviation (SD). *P* values of less than 0.05 were considered significant (ns: not significant, *: *p* < 0.05, **: *p* < 0.01, ***: *p* < 0.001, ****: *p* < 0.0001).

## Supplementary Information

Supplementary Legends.

Supplementary Figure 1.

Supplementary Figure 2.

Supplementary Figure 3.

Supplementary Table 1.

## Data Availability

The datasets generated and analyzed during the current study are available from the corresponding author on reasonable request.

## Abbreviations

AUC: Area under the curve
CR-POPF: Clinically relevant postoperative pancreatic fistula
CT: Computed tomography
FRS: Fistula risk score
POPF: Postoperative pancreatic fistula
PRV: Pancreatic remnant volume
rFRS: Radiological fistula risk score
SD: Standard deviation
sFRS: Surgical fistula risk score
