# Supplementary material for: The image-based preoperative fistula risk score (preFRS) predicts postoperative pancreatic fistula in patients undergoing pancreatic head resection
Source: Sci Rep. 2022 Mar 8;12:4064. doi: 10.1038/s41598-022-07970-2 (PMC8904506; doi:10.1038/s41598-022-07970-2)
Supplement: Supplementary file 1 — Supplementary Legends. [file 41598_2022_7970_MOESM1_ESM.docx]

## Supplementary Figure Legends

### Supplementary Figure 1

**Suppl. Fig. 1 Image-based evaluation of rFRS and preFRS parameters. (a-b)** Example illustration of risk factor assessment for determination of rFRS in axial reconstructions of preoperative contrast-enhanced CT images. **(a)** Preoperative contrast-enhanced CT image of a 65-year old male patient with a pancreatic head tumor (arrow), consistent with pancreatic adenocarcinoma. The remaining pancreatic parenchyma is atrophic and the pancreatic duct (arrowhead) measures 8.5 mm in diameter. **(b)** Preoperative contrast-enhanced CT image of a 68-year old female patient with a pancreatic head tumor (arrow), consistent with pancreatic adenocarcinoma. The remaining pancreatic parenchyma is normal and the pancreatic duct (arrowhead) measures 2.1 mm in diameter.

### Supplementary Figure 2

**Suppl. Fig. 2** **Image-based evaluation of pancreatic texture for rFRS and preFRS.** Example illustration of atrophic and normal pancreatic remnant parenchyma (arrowheads) in axial reconstructions of preoperative contrast-enhanced CT images.

### Supplementary Figure 3

**Suppl. Fig. 3** **Validation of CT-based PRV estimation.** Points represent individual patients, colors represent manifestation of CR-POPF (grey: no CR-POPF, black: CR-POPF), line represents simple linear regression line. Abbreviations: clinically relevant postoperative pancreatic fistula (CR-POPF), pancreatic remnant volume (PRV).

## 
