# Supplementary figures and images for: The image-based preoperative fistula risk score (preFRS) predicts postoperative pancreatic fistula in patients undergoing pancreatic head resection
Source: Sci Rep. 2022 Mar 8;12:4064. doi: 10.1038/s41598-022-07970-2 (PMC8904506; doi:10.1038/s41598-022-07970-2)

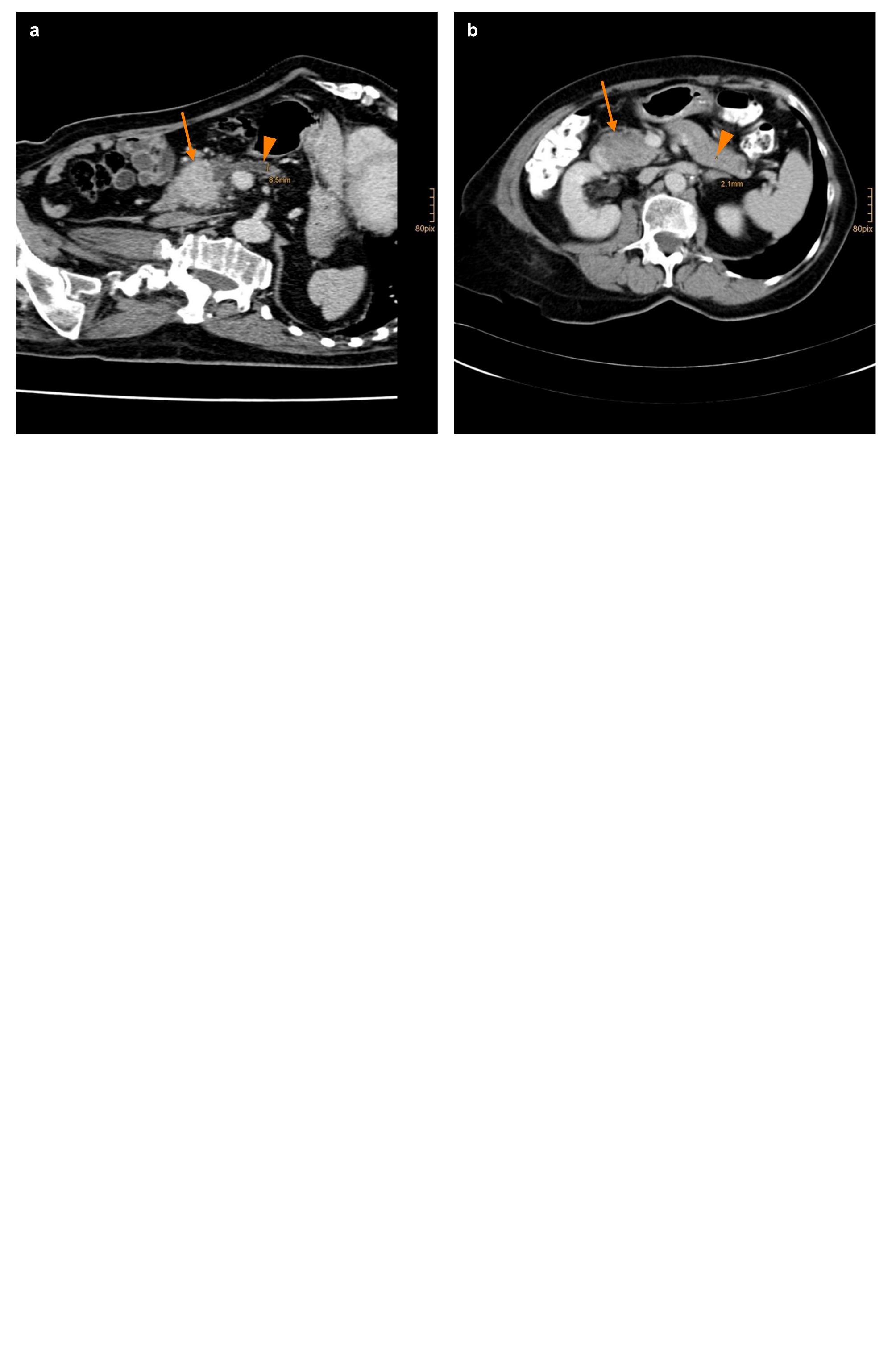

Supplement: Supplementary file 2 — Supplementary Figure 1. [file 41598_2022_7970_MOESM2_ESM.tif]

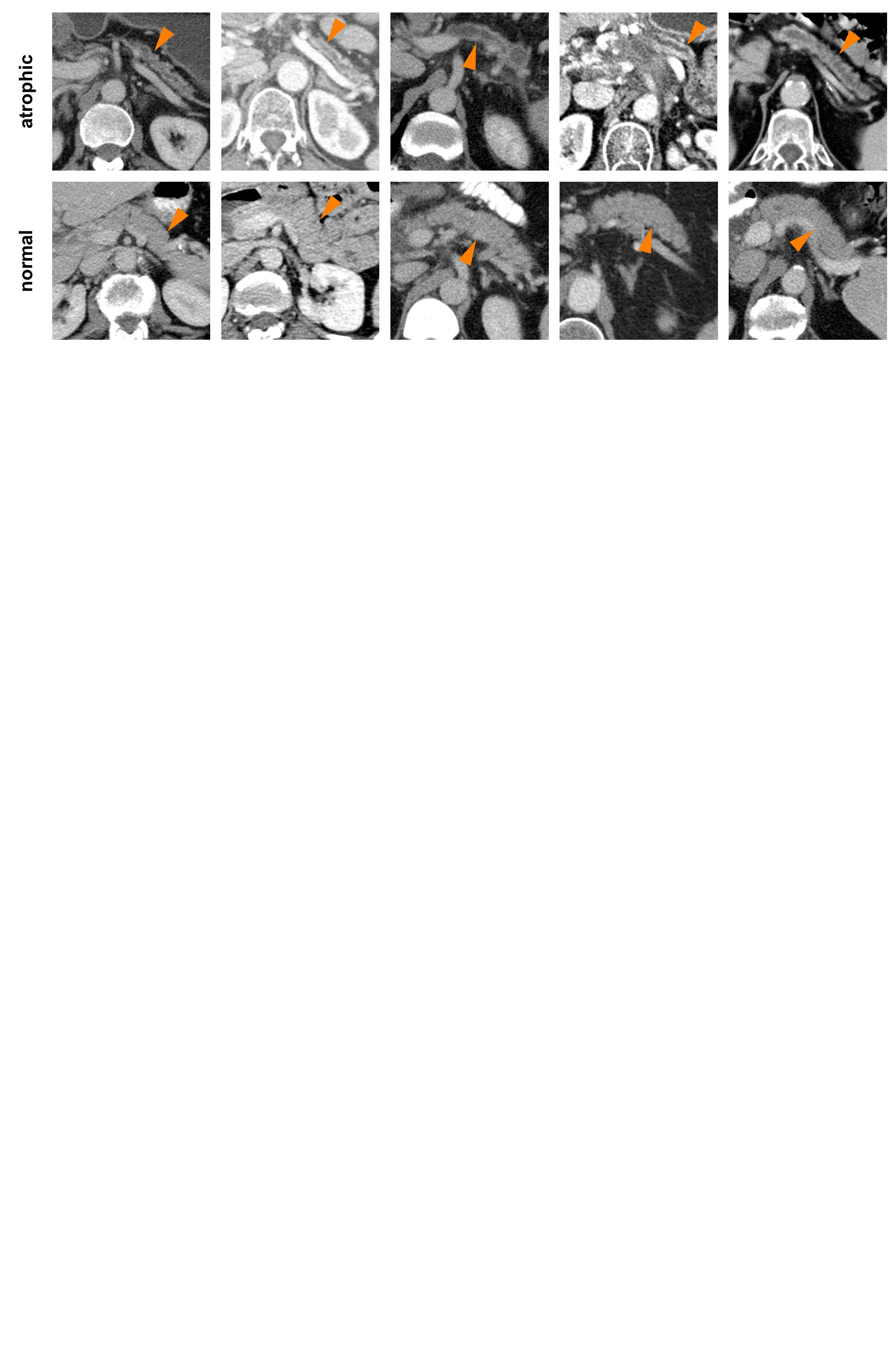

Supplement: Supplementary file 3 — Supplementary Figure 2. [file 41598_2022_7970_MOESM3_ESM.tif]

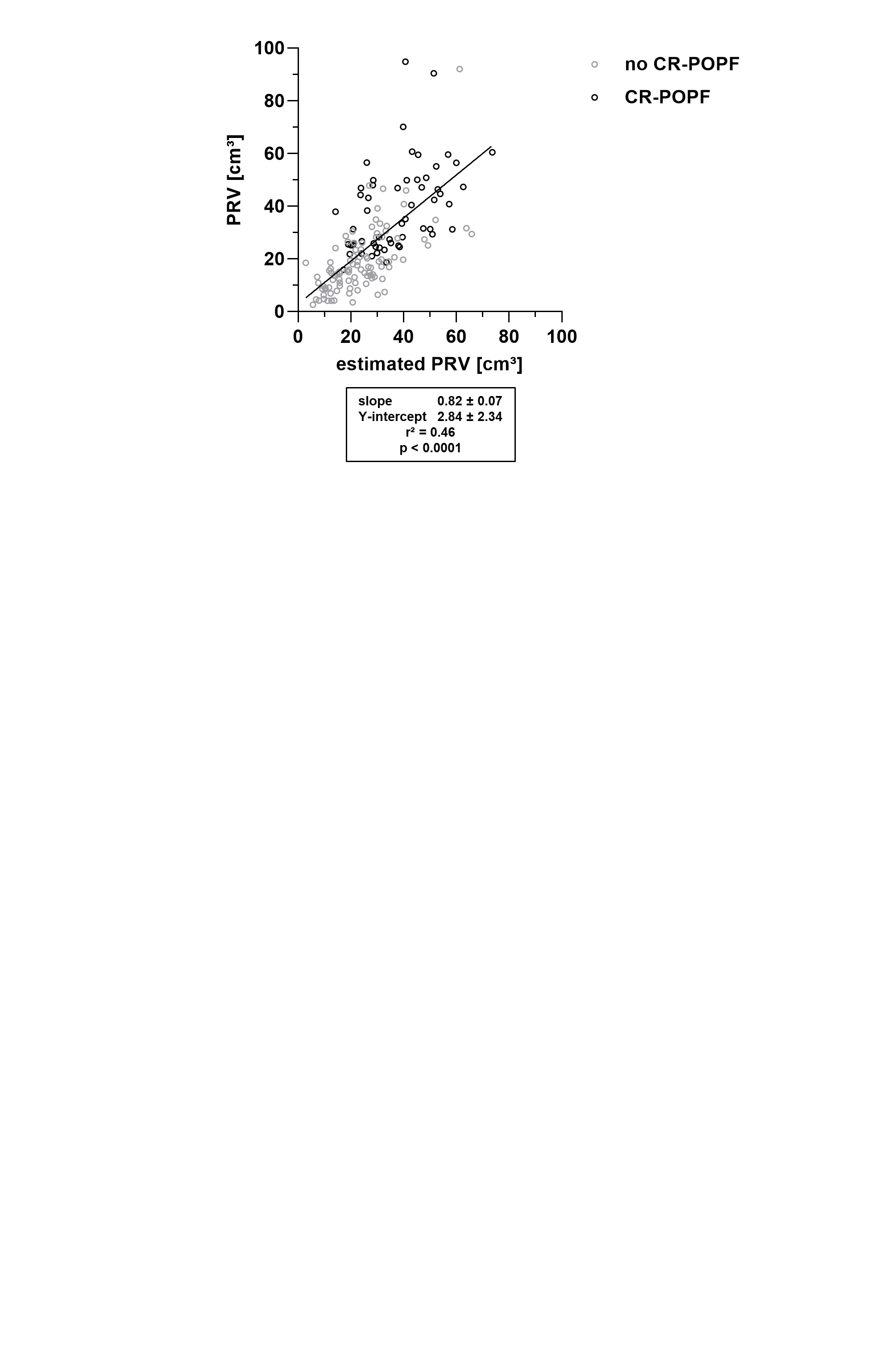

Supplement: Supplementary file 4 — Supplementary Figure 3. [file 41598_2022_7970_MOESM4_ESM.tif]
