# Supplementary material for: The image-based preoperative fistula risk score (preFRS) predicts postoperative pancreatic fistula in patients undergoing pancreatic head resection
Source: Sci Rep. 2022 Mar 8;12:4064. doi: 10.1038/s41598-022-07970-2 (PMC8904506; doi:10.1038/s41598-022-07970-2)
Supplement: Supplementary file 5 — Supplementary Table 1. [file 41598_2022_7970_MOESM5_ESM.docx]

## Supplementary Table

### Supplementary Table 1

**Suppl. Tab. 1** **Prediction performance of the developed risk models for sFRS and rFRS risk factors using leave-one-out cross-validation.** Furthermore, the association of the individual parameters included in the trained models were tested in the entire patient cohort. Abbreviations: area under the curve of the receiver-operator characteristic curve (AUC), pancreatic remnant volume (PRV).

|  | **Risk factor** | **p-value** | **Prediction performance** |
| --- | --- | --- | --- |
| **sFRS** |  |  | AUC (training): 0.85  AUC (validation): 0.80  F1 score (training): 0.64  F1 score (validation): 0.63 |
|  | **Pancreatic texture** | < 0.001 |  |
|  |  |  |  |
|  | **Pathology** | < 0.001 |  |
|  |  |  |  |
|  | **Pancreatic duct diameter** | < 0.001 |  |
|  |  |  |  |
| **rFRS** |  |  | AUC (training): 0.82  AUC (validation): 0.81  F1 score (training): 0.56  F1 score (validation): 0.54 |
|  | **Pancreatic texture** | < 0.001 |  |
|  |  |  |  |
|  | **Pathology** | 0.004 |  |
|  |  |  |  |
|  | **Pancreatic duct diameter** | < 0.001 |  |
|  |  |  |  |
| **rFRS including additional image-based parameters** |  |  |  |
|  | **Estimated PRV [cm^3^]** | < 0.001 | AUC (training): 0.85  AUC (validation): 0.83  F1 score (training): 0.58  F1 score (validation): 0.56 |
|  |  |  |  |
|  |  |  |  |
|  | **Normalized pancreatic density** | 0.49 | AUC (training): 0.85  AUC (validation): 0.83  F1 score (training): 0.63  F1 score (validation): 0.56 |
|  |  |  |  |
| **rFRS including additional**  **image-based and clinical parameters** | **Age**  **BMI**  **Diabetes mellitus before surgery**  **Sex**  **Surgery method (PPPD/Whipple)** | 0.36  < 0.001  0.011  0.62  0.18 | AUC (training): 0.87  AUC (validation): 0.83  F1 score (training): 0.63  F1 score (validation): 0.53 |
